# When Seed and Soil Theory Meets Chicken or Egg Theory in Cancer Metastasis

**DOI:** 10.4172/2168-9652.1000e131

**Published:** 2015-02-02

**Authors:** Meenu Jain, Ali S Arbab, BR Achyut

**Affiliations:** Tumor Angiogenesis Lab, Biochemistry and Molecular Biology Department, Cancer Center, Georgia Regents University, Augusta, GA 30912, USA

Cancer metastasis has been a serious problem since decades. Seed and soil hypothesis of metastasis remains true and all the metastatic tumors follow this nature’s law [1,2]. Advance metastasis or multi-organ metastasis is difficult to treat due to multi-organ dysfunction. One of the major issues in metastasis is that diagnosis occurs at the advanced stages. Secondly, we have not understood the complete mechanisms so well so far due to intricate nature of metastasis. In metastasis, seed (tumor cell) migrates to the soil (distant organs, e.g. lung, liver, brain, and bone). Several experimental studies have been done that suggested the role of bone marrow derived progenitor cells [3] (e.g. CD11b+ [4] and VEGFR1+ [5] cells) in the initiation of metastasis. Several chemokines, such as SDF-1, TNF-α, TGF-β and VEGF-A influence the recruitment of different cell types to pre-metastatic sites resulting into increased expression of specific molecules in the niche like S100A8, S100A9, lysyl oxidase (LOX), fibronectin, MMP9 and MMP2 in the initiation of premetastatic niche [6,7], which are bonafide candidates of therapeutic targeting [8]. In addition, tumor induced hypoxia has been shown to promote the premetastatic niche formation by recruiting CD11b+/Ly6Cmed/Ly6G+ cells [9] and producing LOX [10].

Recently, much attention has been given to the tumor-derived exosomes or micro vesicles that carry almost every essential cellular macromolecule and has signals to polarize cells in the tumor microenvironment and create premetastatic niche in the distant organs, before the seed (tumor cell) arrives [11,12]. Exosomes derived from melanomas were shown to educate pro-metastatic progenitor cells in the bone marrow [13]. Renal-carcinoma-derived exosomes were found to promote angiogenesis in lung tumor metastases [14]. In addition, using murine mammary carcinoma demonstrated that, tumor-derived microvesicles use osteopontin to mobilize pro-angiogenic cells from the bone marrow [15]. Surprisingly, exosomes perform cell independent miRNA biogenesis to promote tumorigenesis and metastasis [16]. Firstly, tumor derived exosomes has pro-angiogenic functions that helps tumor in building required vasculature for tumor growth. For example, Yoon et al. [17] investigated pro-angiogenic role of tumor-secreted exosomes by showing Egr-1 activation in endothelial cells through ERK1/2 and JNK signaling pathways and endothelial cell migration, which was facilitated by the tumor cell derived extracellular vesicles. On the other hand, tumor derived exosomes involved in the destruction of vasculature integrity for metastasis. For example, miR-105, which is characteristically expressed and secreted by metastatic breast cancer cells, is reported as a potent regulator of tumor cell migration through targeting the tight junction protein ZO-1 via exosomes. Tumor cell secreted exosomes deliver miR-105 to the site of endothelial monolayers that efficiently destroys tight junctions and hence the integrity of barriers against metastasis [18].

Although exosomes have attracted much attention and are considered as a bonafide targets for cancer therapy, their roles in tumor metastasis is poorly investigated. In addition, technologies and methods to study exosomes are growing day by day. It is possible that tumor cell exosomes are delivered to the distant organs that manipulates host environment before any immune cells or chemokine. However, what initiates tumor cell migration to the distant organs, remains unclear, i.e. chicken comes first or egg and warrants further investigations.
